# Fast-Track Signaling: A Non-Adiabatic Photoactivation Pathway in Plant Cryptochromes

**DOI:** 10.1021/acscentsci.5c01100

**Published:** 2025-07-01

**Authors:** Jorim M. Kornblueh, Ilia A. Solov’yov

**Affiliations:** † Institute of Physics, 11233 Carl von Ossietzky Universität Oldenburg, Carl-von-Ossietzky Straße 9-11, 26129 Oldenburg, Germany; ‡ Research Centre for Neurosensory Science, Carl von Ossietzky Universität Oldenburg, Carl-von-Ossietzky-Str. 9-11, 26129 Oldenburg, Germany; § Center for Nanoscale Dynamics (CENAD), Carl von Ossietzky Universität Oldenburg, Ammerländer Heerstr. 114-118, 26129 Oldenburg, Germany

## Abstract

A non-adiabatic electron transfer route in cryptochromes expands understanding of flavin photoactivation and light sensing in biology.

## Graphical abstract

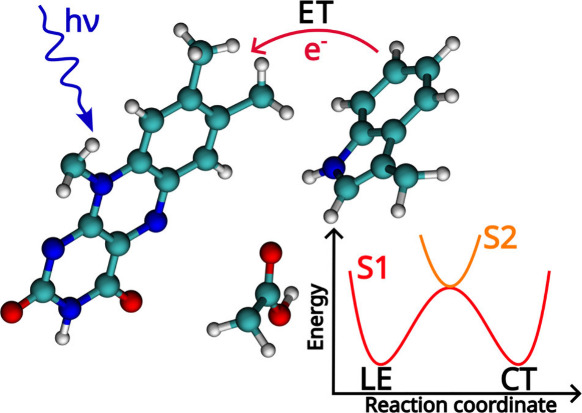

Cryptochromes are blue-light-sensitive flavoproteins that mediate essential biological functions across the kingdoms of life.^1^ In plants, they regulate developmental transitions such as photomorphogenesis and flowering; in animals, they help govern circadian rhythms. Notably, growing evidence supports a role for cryptochromes in magnetoreception—the ability of particular species, including migratory birds, to detect Earth’s magnetic field using light-dependent biochemical signals.^2^ This hypothesis hinges on the formation of spin-correlated radical pairs initiated by photoinduced electron transfer (ET) along a conserved tryptophan chain following flavin adenine dinucleotide (FAD) excitation, bound at the active core of the protein.^3,4^ Despite this general framework, mechanistic uncertainties remain, especially regarding the initial steps following photoexcitation.^5^ Do classical, adiabatic relaxation models best describe these early ET events, or do ultrafast nonadiabatic transitions, long recognized in small-molecule photophysics, also play an active role in protein environments?

In this issue of *ACS Central Science*, Costa and Liang provide evidence for nonadiabatic effects in the protein environment.

Using a rigorous combination of nonadiabatic *ab initio* quantum mechanics/molecular mechanics (QM/MM) dynamics and multireference electronic structure methods, they explore the excited-state behavior of *Arabidopsis thaliana* cryptochrome 1 (AtCry1), a prototypical plant cryptochrome.^6^ Their findings complement the traditional assumptions about ET in proteins and introduce a new quantum dynamical pathway that expands the theoretical landscape of cryptochrome photoactivation.

The key discovery of Costa and Liang is identifying a nonadiabatic ET pathway operating in AtCry1 on a sub-10 fs time scale from the transient bright S2 excited state to a charge-transfer (CT) state on the S1 energy surface (Figure 1C). This transition is mediated by a conical intersection, an archetypal feature of ultrafast photochemistry, and leads to the rapid formation of the [FAD^•–^–W400^•+^] radical pair; here an electron is donated by the W400 residue to the flavin cofactor, noncovalently bound inside the protein matrix (Figure 1A). This fast-track ET mechanism elegantly circumvents kinetic bottlenecks associated with adiabatic relaxation through intermediate locally excited (LE) states. Costa and Liang’s approach is exemplary in its methodological depth. Using the *ab initio* multiple spawning (AIMS) algorithm at the complete active space self-consistent field (CASSCF) level of theory it enables the treatment of both electronic and nuclear degrees of freedom in a fully nonadiabatic regime. This approach is a significant advance over prior work, which relied on adiabatic surface hopping or static Marcus-like models. Furthermore, the authors integrate realistic protein environments through QM/MM coupling, accurately representing the electrostatic and structural constraints that modulate ET in biological systems.

**Figure 1:**
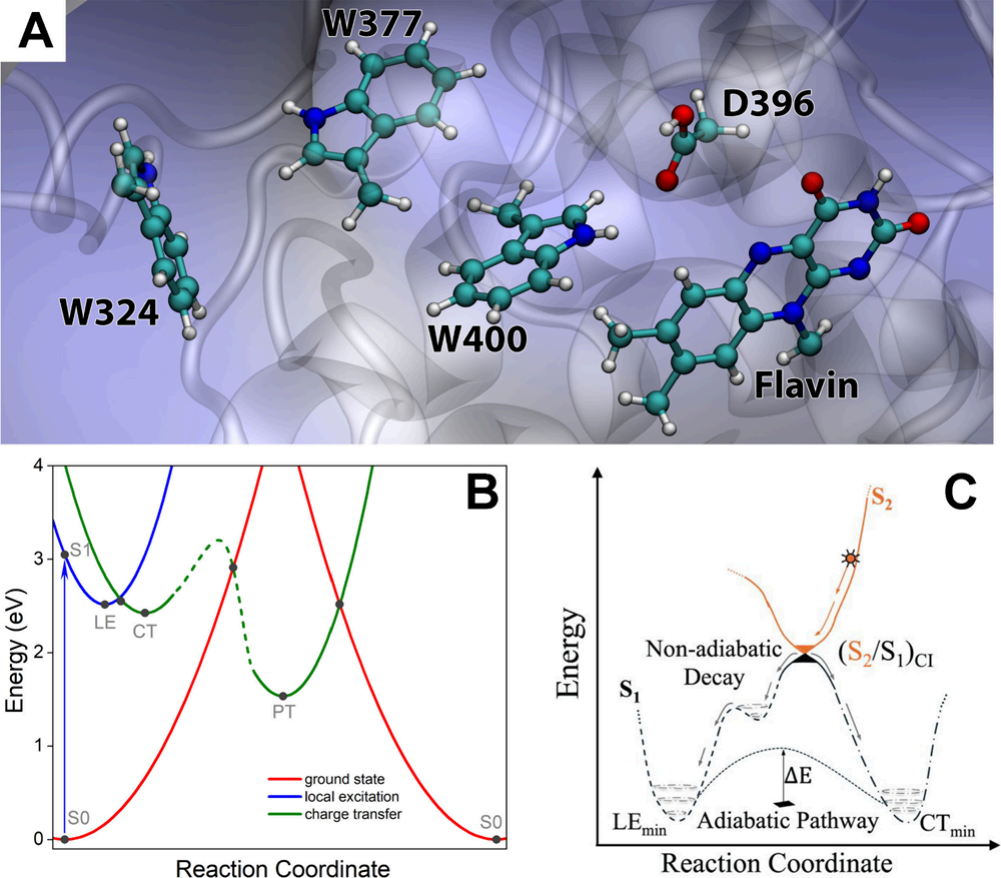
(A) Active site of AtCry1 involving the flavin cofactor and the W400, W377, W324, and D396 residues. (B) Calculated potential energy profiles of the key electronic states describing AtCry1 photoactivation. The energy of the oxidized flavin (ground state) is shown in red, locally excited flavin in blue, and the [FAD^•–^–W400^•+^] radical pair state (charge transfer), in green. Solid circles represent computed energies for the optimized ground state (S0), flavin excitation (S1) and optimized local excitation (LE), optimized charge transfer (CT) and the stabilized CT state after flavin protonation by the D396 residue (PT). The dashed line indicates a possible barrier between the CT and the PT states. Figure adapted with permission from ref 7. Copyright 2012 American Chemical Society. (C) Schematic representation of the nonadiabatic and adiabatic ET mechanisms in AtCry1. Reproduced from ref 6. Available under a CC-BY 4.0 license. Copyright 2025 Gustavo J. Costa, Ruibin Liang.

Costa and Liang’s simulations partially resolve the longstanding discrepancy in the field concerning the mismatch between theoretical ET rates and experimental observations.

Earlier experimental studies of plant cryptochromes^8^ predicted ET to occur rapidly (with a rate constant of 0.4 ps^–1^) following light absorption. However, adiabatic simulations could not consistently reproduce this result directly or yielded incomplete radical pair formation. Costa and Liang identified a second, lower-energy LE minimum on the S1 surface that acts as a kinetic trap. Adiabatic trajectories often become sequestered in this basin, failing to access the CT state promptly. The ET cases in the nonadiabatic regime yielded ultrafast radical pair formation, where even the experimental rates were surpassed. Therefore, combining adiabatic and nonadiabatic processes may produce the correct picture.

While this work marks a clear advance in computational photobiology, its direct relevance to magnetoreception, an aspect of cryptochrome function that remains not fully understood, appears limited. The study focuses on a plant cryptochrome that is structurally different from the cryptochromes used by higher animals for geomagnetic field sensing. Notably, AtCry1 contains only three tryptophan residues in its ET chain (Figure 1A), whereas avian cryptochromes possess a four-tryptophan tetrad. This extension has been functionally implicated in prolonging radical pair lifetimes, which is directly related to weak magnetic field sensitivity.^2,9^ Furthermore, the aspartic acid residue (D396) that facilitates FAD protonation in AtCry1 ^7^ (Figure 1A)^3^ is absent in animal cryptochromes, leading to distinct redox and structural dynamics.

Prior work has thoroughly examined the role of structural differences between cryptochromes from different organisms and their mutants in modulating ET and magnetic field sensitivity. For instance, studies by Xu et al.^2^ and Timmer et al.^5^ on avian cryptochromes revealed distinct ET kinetics and suggested functional specialization of the Trp tetrad for magnetoreception. Additionally, spectroscopic and theoretical studies have shown that the geometry and polarity of the protein matrix and its environment can significantly influence radical pair formation and magnetic sensitivity in cryptochromes.^3,10^

Costa and Liang’s work builds upon a foundation laid over a decade ago.

An earlier investigation^7^ utilized QM/MM methods involving CASSCF and XMCQDPT2 (extended multiconfigurational quasidegenerate perturbation theory) approaches to study AtCry1 photochemistry. The study resolved the key steps of the ET process (Figure 1B), including radical pair formation and the effects of protein electrostatics. Although the active space in that work was more limited and nonadiabatic dynamics were not explored, the fundamental mechanistic picture was broadly consistent. The new findings by Costa and Liang can thus be viewed as an important refinement of the established model rather than a conceptual redefinition enabled by improved computational tools and deeper configurational sampling.

That said, the significance of Costa and Liang’s work should not be understated.

Their demonstration that nonadiabatic transitions can be present in large, electrostatically complex proteins challenges the traditional view that ultrafast photochemistry is confined to small organic systems. It opens the door to reexamining other biological photoreceptors, such as photolyases and animal cryptochromes, for similar dynamical features.

Their work also lays a computational foundation for future studies that could address structurally homologous animal proteins under realistic conditions.

The study raises additional exciting questions about the role of S2 excitation in cryptochrome function. Historically, the S2 excited state has been considered biologically irrelevant due to its low oscillator strength. The findings of Costa and Liang, however, suggest that this assumption is not necessarily always valid, as their calculations indicate that S2 excitation could become kinetically significant, mainly if it provides access to otherwise inaccessible CT pathways.

In conclusion, Costa and Liang’s work exemplifies the power of quantum dynamical simulations to clarify mechanistic ambiguities in biological ET. While its direct relevance to magnetoreception is limited, the study delivers a rigorous and novel account of ultrafast nonadiabatic electron transfer in a plant cryptochrome, introduces new theoretical insight, and prompts a rethinking of long-held assumptions in photoreceptor biology. It is a technically superb and conceptually valuable contribution to the field that will likely inspire further inquiry into the quantum foundations of biological sensing.
